# Targeting angiogenesis in gastrointestinal tumors: strategies from vascular disruption to vascular normalization and promotion strategies angiogenesis strategies in GI tumor therapy

**DOI:** 10.3389/fimmu.2025.1550752

**Published:** 2025-04-22

**Authors:** Jiajia Li, Zhengrui Li, Keliang Wang

**Affiliations:** ^1^ Department of Gastroenterology, Ningbo No.2 Hospital, Ningbo, Zhejiang, China; ^2^ School of Rehabilitation Medicine, Binzhou Medical University, Yantai, Shandong, China; ^3^ Department of Oral and Maxillofacial-Head and Neck Oncology, Shanghai Ninth People’s Hospital, Shanghai Jiao Tong University School of Medicine, Shanghai, China

**Keywords:** angiogenesis, gastrointestinal tumors, vessel disruption, therapeutic strategies, molecular mechanisms, clinical applications

## Abstract

Angiogenesis plays a critical role in the progression of gastrointestinal (GI) tumors, making it an important therapeutic target. This review explores recent advancements in targeting angiogenesis for GI tumor therapy, highlighting strategies that range from vascular disruption to vascular promotion. The biological foundation of tumor angiogenesis is discussed, with a focus on the molecular mechanisms that regulate this process, including key players such as VEGF, HIFs, and non-coding RNAs. Current therapeutic strategies, including anti-angiogenic agents, vascular normalization approaches, and emerging vascular promotion therapies, are analyzed for their clinical applications and limitations. Additionally, the review examines combination strategies that integrate anti-angiogenic therapy with chemotherapy, immunotherapy, and other modalities to enhance efficacy and overcome resistance. Despite significant progress, challenges such as drug resistance, tumor heterogeneity, and adverse effects remain. Future research directions emphasize the discovery of novel molecular targets, development of personalized treatments, and innovative combination therapies to optimize outcomes for patients with GI tumors. This comprehensive review provides a foundation for advancing angiogenesis-targeted therapies in GI cancer treatment.

## 1 Introduction

**Figure 1:**
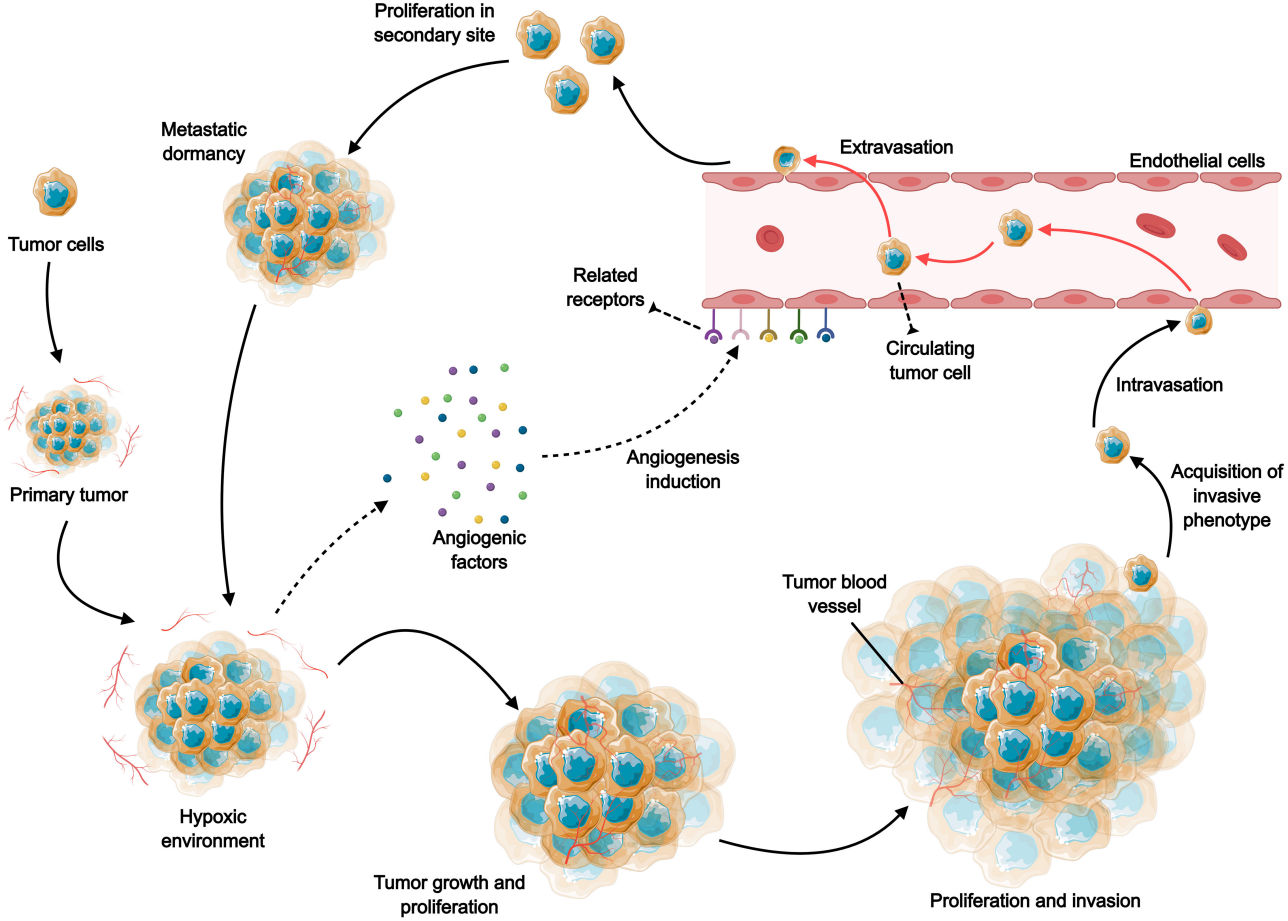
The role of angiogenesis in tumor growth, invasion and metastasis. Rapid tumor expansion leads to reduced oxygen supply, and the resulting tumor microenvironment stimulates excessive angiogenesis by increasing various pro-angiogenic factors, including VEGF, PDGF, FGF, and angiopoietin. Subsequently, the neovascularization facilitates the transport of oxygen and nutrients, further supporting the survival, growth and proliferation of tumor cells. As tumor cells develop a more aggressive phenotype, they continue to proliferate, spread and induce angiogenesis, and tumor cells invade and metastasize through the blood circulation to distant tissues.

Gastrointestinal (GI) tumors are among the most prevalent cancers globally, presenting significant challenges in treatment and management (1). The heterogeneity of gastrointestinal tumors is multifaceted, involving histological types, tumor microenvironment (TME), and genetic mutations. The complexity of gastrointestinal tumors stems from their diverse histological types, varying biological behaviors, and the unique microenvironment in which they develop (2). This heterogeneity complicates the treatment landscape, as standard therapies may not be equally effective across different tumor types. The increasing incidence of these tumors, combined with their often-late presentation and aggressive nature, necessitates the exploration of innovative therapeutic strategies. One promising area of research is the targeting of tumor angiogenesis, a process critical for tumor growth, invasion, and metastasis (**Figure 1**). Angiogenesis, the formation of new blood vessels from pre-existing ones, plays a pivotal role in supplying nutrients and oxygen to tumors, thereby facilitating their progression (3, 4).

Tumor angiogenesis is a complex biological process regulated by a balance between pro-angiogenic and anti-angiogenic factors. In the context of gastrointestinal tumors, this balance is often disrupted, leading to excessive angiogenesis that supports tumor growth and metastasis. The vascular endothelial growth factor (VEGF) is one of the most studied pro-angiogenic factors, and its overexpression is frequently associated with poor prognosis in various cancers, including gastrointestinal tumors (5). Targeting angiogenesis has emerged as a viable therapeutic strategy, with several anti-angiogenic agents currently in clinical use or under investigation (6). These agents aim to inhibit the formation of new blood vessels, thereby starving the tumor of essential nutrients and oxygen (7). However, the efficacy of these treatments can be limited by the development of resistance, necessitating a deeper understanding of the underlying mechanisms of angiogenesis and the tumor microenvironment (8).

Recent advancements in the understanding of tumor angiogenesis have led to the development of innovative strategies that not only inhibit vessel formation but also aim to normalize the abnormal tumor vasculature. This normalization can enhance the delivery and efficacy of concurrent therapies, such as chemotherapy and immunotherapy (9). Approaches that combine anti-angiogenic therapy with other treatment modalities are being explored to overcome resistance and improve patient outcomes (10). Furthermore, the identification of biomarkers associated with angiogenesis may help in selecting patients who are most likely to benefit from these targeted therapies (11, 12). The dual strategies of disrupting existing blood vessel formation and promoting a more normalized vascular structure present a promising avenue for enhancing the effectiveness of gastrointestinal cancer treatments (13).

In addition to traditional anti-angiogenic therapies, recent studies have highlighted the potential of novel agents and combination therapies that target multiple pathways involved in angiogenesis. For instance, the use of natural products with anti-angiogenic properties has gained attention due to their lower toxicity profiles and ability to target multiple signaling pathways simultaneously. Compounds such as Britanin have shown promise in preclinical studies for their ability to modulate angiogenesis and inhibit tumor growth in various gastrointestinal cancers (14). Moreover, ongoing research into the role of long non-coding RNAs and microRNAs in regulating angiogenic processes offers new insights into potential therapeutic targets. These molecular players may serve as both biomarkers and therapeutic targets, paving the way for more personalized treatment strategies in the management of gastrointestinal tumors (15, 16).

In conclusion, targeting angiogenesis presents a multifaceted approach to the treatment of gastrointestinal tumors. The interplay between tumor angiogenesis and the tumor microenvironment is complex, and a comprehensive understanding of these interactions is essential for the development of effective therapies(Li, 17). As research continues to unveil the intricacies of angiogenesis in gastrointestinal tumors, the integration of novel therapeutic strategies alongside established treatments holds the promise of improving patient outcomes and addressing the challenges posed by these aggressive malignancies. Future studies should focus on elucidating the molecular mechanisms governing angiogenesis and the potential for combining various therapeutic modalities to enhance treatment efficacy and overcome resistance.

## 2 Biological basis of angiogenesis in gastrointestinal tumors

### 2.1 Definition and importance of tumor angiogenesis

Tumor angiogenesis refers to the physiological process through which new blood vessels form from pre-existing ones, a critical mechanism that supports tumor growth and metastasis. In the context of gastrointestinal tumors, including gastric and colorectal cancers, angiogenesis is essential for providing the necessary nutrients and oxygen to rapidly proliferating cancer cells (9). It is widely recognized as a hallmark of cancer progression and is closely associated with tumor aggressiveness and poor prognosis (7). Tumors can only grow beyond a size of 2 mm through the establishment of new blood vessels, which are crucial for tumor survival and expansion (18).

Angiogenesis is not simply a passive response to tumor growth but is actively regulated by a complex interplay of pro-angiogenic and anti-angiogenic factors. Disruption of this balance can lead to pathological angiogenesis, characterized by abnormal blood vessels that contribute to tumor metastasis and resistance to therapies (19). Thus, understanding the molecular mechanisms underlying angiogenesis is vital for the development of targeted therapies aimed at inhibiting this process in GI cancers (20).

### 2.2 Molecular mechanisms of angiogenesis in gastrointestinal tumors

The molecular mechanisms underlying angiogenesis in gastrointestinal tumors are multifaceted and involve several keys signaling pathways and factors. Central to this process is the VEGF family, which plays a pivotal role in promoting endothelial cell proliferation, migration, and survival (21). Other important players include hypoxia-inducible factors (HIFs), fibroblast growth factors (FGFs), and matrix metalloproteinases (MMPs), which collectively orchestrate the angiogenic response in the tumor microenvironment (22).

**Figure 2:**
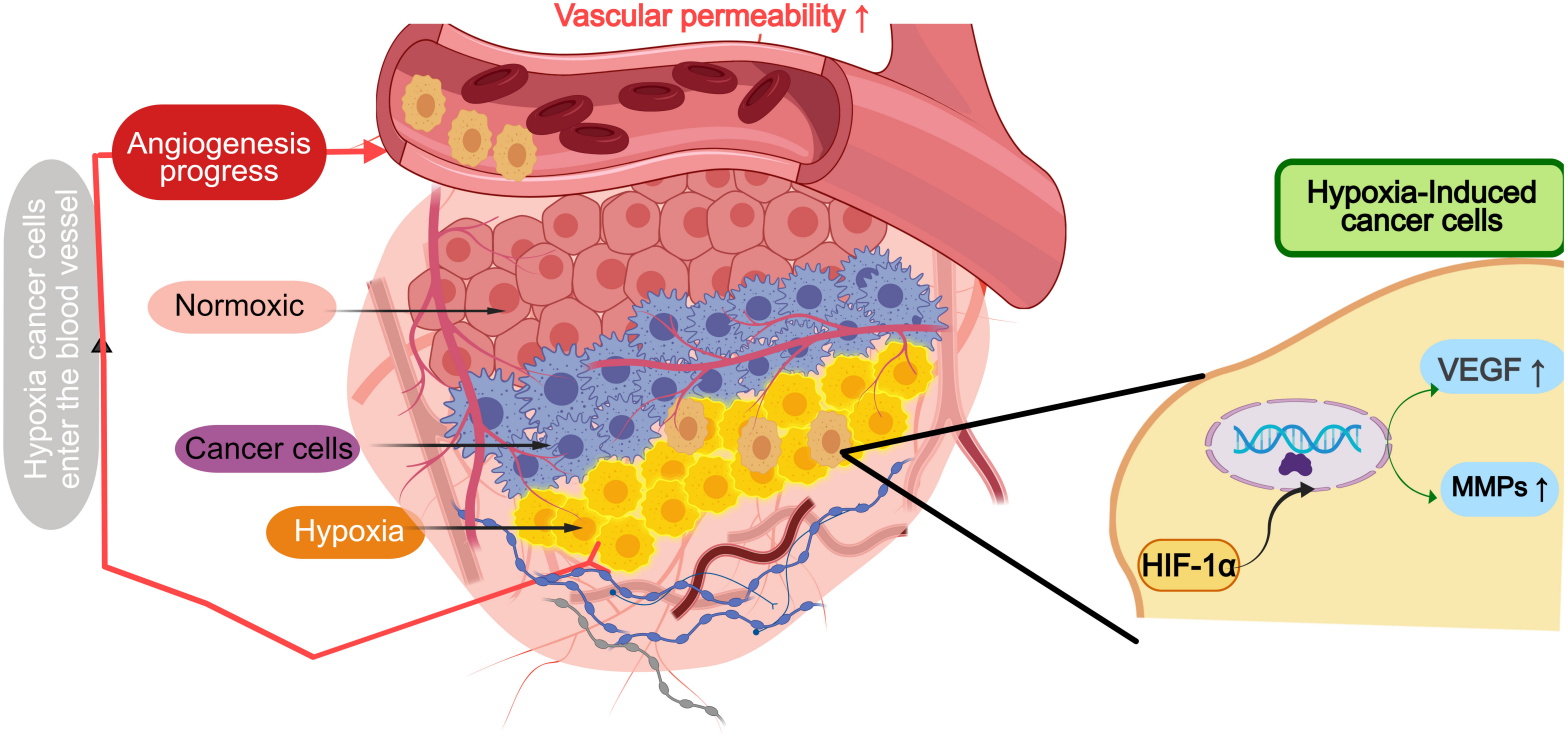
Angiogenesis in hypoxic cancer microenvironment of gastrointestinal tumors. HIF-1α stabilizes and promotes transcription of VEGF, MMPs and other factors under hypoxic conditions, increases vascular permeability and promotes abnormal formation of tumor vessels.

In GI tumors, dysregulation of angiogenic factors plays a crucial role in tumor progression and metastasis (**Figure 2**). Under hypoxic conditions, HIF-1α stabilizes and promotes VEGF transcription, increasing vascular permeability and fostering abnormal blood vessel formation (23). In gastric cancer, hypoxia-driven stabilization of HIF-1α results in the upregulation of VEGF and matrix metalloproteinase 9 (MMP9), which not only enhances tumor invasion but also contributes to the dysregulated vasculature commonly observed in these cancers (24). Additionally, VEGF amplification in colorectal cancer is regulated by Wnt/β-catenin signaling, further exacerbating the aberrant angiogenesis and vascular dysfunction in these tumors (25).

Additionally, long non-coding RNAs (lncRNAs) and microRNAs (miRNAs) have emerged as critical regulators of angiogenesis in GI tumors. LncRNAs like PVT1 sponge miRNAs to upregulate angiogenic factors like VEGFA in hepatocellular carcinoma (26). Similarly, miRNAs such as miR-26a play a crucial role in modulating the expression of VEGF and other angiogenic factors, influencing endothelial cell behavior, and regulating the tumor vasculature (27). This interplay underscores the complexity of angiogenesis in GI tumors and highlights the need for a deeper understanding of these mechanisms (28).

The dysregulation of these molecular signals not only facilitates tumor growth but also contributes to the formation of an irregular and dysfunctional vascular network. These abnormal vessels are typically leaky and inefficient, posing significant challenges for drug delivery and immune cell infiltration, which complicates therapeutic interventions (29). Moreover, the presence of abnormal angiogenesis often correlates with poor prognosis, emphasizing the critical need for targeted therapies aimed at normalizing the tumor vasculature and addressing the molecular underpinnings of angiogenesis in GI cancers.

### 2.3 Balance of angiogenic and anti-angiogenic factors

The balance between pro-angiogenic and anti-angiogenic factors is crucial in regulating angiogenesis within gastrointestinal tumors. Pro-angiogenic factors, such as VEGF and FGF, promote the formation of new blood vessels, while anti-angiogenic factors, such as thrombospondin-1 (TSP-1), inhibit this process (30). In GI cancers, this balance is often disrupted in favor of pro-angiogenic signals, leading to excessive angiogenesis that supports tumor growth and metastasis (31). For instance, increased VEGF expression in colorectal cancer is associated with tumor progression and poor clinical outcomes, emphasizing the need for therapeutic strategies targeting this pathway (32).

Recent studies suggest that natural compounds may restore this balance by inhibiting pro-angiogenic pathways, presenting potential therapeutic strategies for managing GI tumors (33). Understanding this balance provides critical insights into tumor biology and opens new avenues for the development of anti-angiogenic therapies aimed at improving patient outcomes in GI cancers (8).

## 3 Vascular disruption strategies

### 3.1 Molecular mechanisms of vascular disruption

The molecular mechanisms underlying vascular disruption are complex and multifaceted, primarily involving the regulation of angiogenesis, which is the formation of new blood vessels from pre-existing ones. Key players in this process include VEGF and its receptors, which are crucial for endothelial cell proliferation, migration, and survival. In cancer, the dysregulation of these pathways often leads to abnormal angiogenesis, contributing to tumor growth and metastasis. For instance, the interaction between VEGF and its receptors can be inhibited by various therapeutic agents, leading to reduced vascular permeability and blood supply to tumors, ultimately impairing their growth (5).

Vascular normalization refers to the process of improving the structural and functional integrity of tumor blood vessels, which are typically irregular, leaky, and dysfunctional. By restoring a more normal vasculature, this strategy aims to enhance the delivery of therapeutic agents, such as chemotherapy or targeted drugs, and to improve the infiltration of immune cells into the tumor. This can significantly increase the efficacy of anti-cancer therapies, as a well-normalized vasculature allows for better perfusion and more efficient distribution of drugs (34, 35). Moreover, vascular normalization can also improve oxygen supply to tumor tissues, mitigating the effects of hypoxia, which often renders cancer cells more resistant to treatment (36). Furthermore, several agents, including anti-VEGF therapies, have been shown to promote vascular normalization by reducing endothelial cell proliferation and encouraging the formation of a more organized vascular structure, which in turn enhances immune cell trafficking and enhances the response to immunotherapy (37, 38). Therefore, while vascular disruption aims to disrupt tumor blood vessels to starve the tumor, vascular normalization holds the potential to optimize drug delivery and immune cell engagement, creating a more favorable environment for cancer treatment.

Moreover, other signaling pathways, such as those involving HIFs and MMPs, also play significant roles in regulating the angiogenic process, especially under hypoxic conditions (39). Recent studies have highlighted the crucial role of metabolic reprogramming in cancer progression, with oxidative stress and nucleotide metabolism being particularly important in gastric and colon cancers (40, 41). These metabolic pathways not only contribute to tumor growth but also influence the tumor microenvironment and immune responses, offering novel therapeutic targets. Understanding these molecular mechanisms is crucial for developing effective vascular disruption strategies, particularly in the context of targeting tumor vasculature to enhance the efficacy of cancer therapies (42).

### 3.2 Clinical applications of vascular disruption drugs

The clinical application of vascular disruption drugs, particularly anti-VEGF agents, has revolutionized the treatment landscape for various malignancies. Anti-VEGF therapies, such as bevacizumab, have been shown to significantly inhibit tumor angiogenesis, leading to reduced tumor growth and improved patient outcomes in conditions like colorectal cancer and glioblastoma (43–45). These agents work by neutralizing VEGF, thereby preventing its interaction with endothelial cells and disrupting the signaling pathways that promote angiogenesis (46, 47).

In addition to monotherapy, anti-VEGF agents are often used in combination with other treatments, such as chemotherapy and immunotherapy, to enhance therapeutic efficacy (48, 49). However, the clinical use of these drugs is not without challenges, including the development of resistance and adverse effects, such as hypertension and proteinuria, which necessitate careful patient monitoring and management (50, 51). Despite these challenges, ongoing research continues to explore novel anti-VEGF agents and combination therapies to optimize treatment outcomes for patients with vascular-dependent tumors (52–54).

However, the widespread use of chemotherapy for advanced gastric cancer has led to increasing cases of drug resistance. Liu et al. provide an in-depth exploration of the mechanisms behind this resistance, focusing on aspects such as tumor microenvironment and non-coding RNA regulation, which could be pivotal in overcoming the limitations of current therapies (55). As resistance to anti-angiogenic therapies and chemotherapy continues to emerge, understanding these mechanisms is essential for improving the efficacy of vascular disruption drugs in gastric cancer therapy.

Emerging next-generation anti-angiogenic agents are currently under investigation to address the limitations of existing therapies. For instance, Apatinib, a selective VEGFR-2 inhibitor, has shown efficacy in preclinical and clinical studies, particularly in gastric cancer, by inhibiting VEGF signaling pathways and suppressing tumor angiogenesis (56, 57). Additionally, exosome miRNA-126, which targets PIK3R2, has shown promise in preclinical models of pancreatic cancer by suppressing angiogenesis, providing a potential non-invasive alternative to traditional anti-VEGF therapies (58, 59). These next-generation agents represent a shift towards more targeted and potentially fewer toxic treatments, offering hope for improving clinical outcomes and overcoming the resistance encountered with earlier anti-angiogenic therapies.

### 3.3 Limitations and challenges of vascular disruption strategies

While vascular disruption strategies, particularly those targeting angiogenesis, have shown promise in cancer therapy, several limitations and challenges persist. One significant challenge is the development of resistance to anti-VEGF therapies, which can occur through various mechanisms, including the upregulation of alternative angiogenic pathways, such as angiopoietin-2 (60). Additionally, the transient nature of the effects of anti-angiogenic agents can lead to tumor regrowth and metastasis once the treatment is withdrawn (7). Furthermore, the heterogeneity of tumor vasculature complicates the effectiveness of these strategies, as not all tumors respond uniformly to anti-VEGF therapies (61).

Further complicating this issue is the emergence of GI tumor-specific resistance mechanisms. For instance, in colorectal cancer, upregulation of the fibroblast growth factor (FGF) pathway or alterations in tumor microenvironment components can drive resistance to angiogenesis inhibitors (8, 62). These tumor-specific alterations may prevent anti-angiogenic therapies from achieving the desired therapeutic effect (63). Clinical trials have also highlighted the adverse effects associated with anti-VEGF therapies, including cardiovascular complications and impaired wound healing, which can limit their applicability in specific patient populations (60). Addressing these challenges requires a multifaceted approach, including the development of combination therapies that target both angiogenesis and the tumor-specific mechanisms of resistance (4). Furthermore, identifying biomarkers to predict the response of GI tumors to vascular disruption therapies is critical for improving treatment outcomes (64). As research progresses, refining vascular disruption strategies and understanding the GI tumor-specific mechanisms of resistance will be crucial in advancing cancer therapies and improving patient prognosis.

## 4 Vascular promotion strategies

### 4.1 Molecular mechanisms of vascular promotion

Vascular promotion involves a complex interplay of molecular mechanisms that facilitate angiogenesis, the formation of new blood vessels from pre-existing ones. The HIF pathway is also crucial, as it regulates the expression of VEGF and other angiogenic factors in response to low oxygen levels, promoting vascular growth in hypoxic tissues (65). Additionally, lncRNAs have emerged as significant regulators of angiogenesis, influencing various oncogenic pathways and modulating the tumor microenvironment (66). Recent studies have highlighted the role of exosome circRNAs and lncRNAs in mediating cell-to-cell communication within the tumor microenvironment, further underscoring their potential as biomarkers and therapeutic targets in cancer (67). The interplay between these molecular pathways not only enhances our understanding of angiogenesis but also opens avenues for targeted therapies aimed at modulating these processes to improve vascularization in pathological conditions (23).

### 4.2 Research progress on vascular promotion drug

Recent advancements in the development of vascular promotion drugs have shown promising results, particularly in the context of gastrointestinal cancers. Natural compounds such as gallic catechin gallate, astragaloside, and curcumin have demonstrated significant anti-angiogenic effects by inhibiting key signaling pathways, including HIF-1α/VEGF and PI3K/Akt/mTOR (21, 68). Furthermore, the use of anti-VEGF therapies has been explored, with mixed results in clinical settings. While agents like bevacizumab have shown efficacy in certain contexts, their long-term use has raised concerns about potential adverse effects, including drug resistance and enhanced metastasis (69). Innovative approaches, such as the targeting of endothelial Notch signaling pathways, have also been investigated, revealing the potential for new therapeutic strategies that could enhance the efficacy of existing treatments (66). The exploration of exosome lncRNAs as therapeutic agents represents a novel frontier in vascular promotion, with studies indicating their role in facilitating vascular regeneration and enhancing drug delivery in cancer therapy (70). Overall, the ongoing research into vascular promotion drugs highlights the need for a multifaceted approach to optimize therapeutic outcomes in cancer treatment.

### 4.3 Clinical application prospects of vascular promotion strategies

The clinical application of vascular promotion strategies holds significant promise, particularly in enhancing therapeutic efficacy in cancer treatment and tissue regeneration. The integration of angiogenic factors and targeted therapies has shown potential in improving outcomes for patients with gastrointestinal tumors, where aberrant angiogenesis plays a critical role in tumor progression (71). Moreover, the use of exosome therapies, which leverage the natural delivery mechanisms of exosomes to transport therapeutic agents, presents a novel approach to enhance vascularization in ischemic tissues and tumors (72). In the context of regenerative medicine, strategies that promote vascularization are crucial for successful tissue engineering, as adequate blood supply is essential for the survival and integration of transplanted tissues (73). Additionally, the development of biomaterials that can release angiogenic factors in a controlled manner is being explored to enhance bone regeneration and repair in orthopedic applications (74). As research progresses, the clinical implementation of these vascular promotion strategies will likely evolve, aiming to improve patient outcomes through more effective and targeted therapies that address the underlying mechanisms of vascular dysfunction in various diseases (75).

## 5 Combination strategies for anti-angiogenic therapy

### 5.1 Combination of vascular disruption and promotion

The dual approach of combining vascular disruption with promotion has emerged as a promising strategy in anti-angiogenic therapy. This strategy recognizes that while inhibiting angiogenesis can starve tumors of necessary nutrients and oxygen, promoting vascular normalization can improve drug delivery and enhance the efficacy of existing therapies. Research has shown that targeting both pathways can lead to a more balanced tumor microenvironment, potentially reducing the adverse effects associated with single-agent therapies. For instance, studies indicate that the simultaneous administration of anti-angiogenic agents, such as bevacizumab, alongside vascular normalization drugs can mitigate the destructive impact of chemotherapy on tumor vasculature, thereby preserving endothelial integrity and function (76). This approach not only aims to inhibit aberrant vessel growth but also to restore the functionality of existing vessels, which is crucial for effective drug delivery and overall tumor control (77). Furthermore, the interplay between these strategies can be influenced by various factors, including the tumor type and microenvironment, necessitating a tailored approach for optimal outcomes (78).

### 5.2 Integration of anti-angiogenic therapy with other treatment modalities

**Figure 3:**
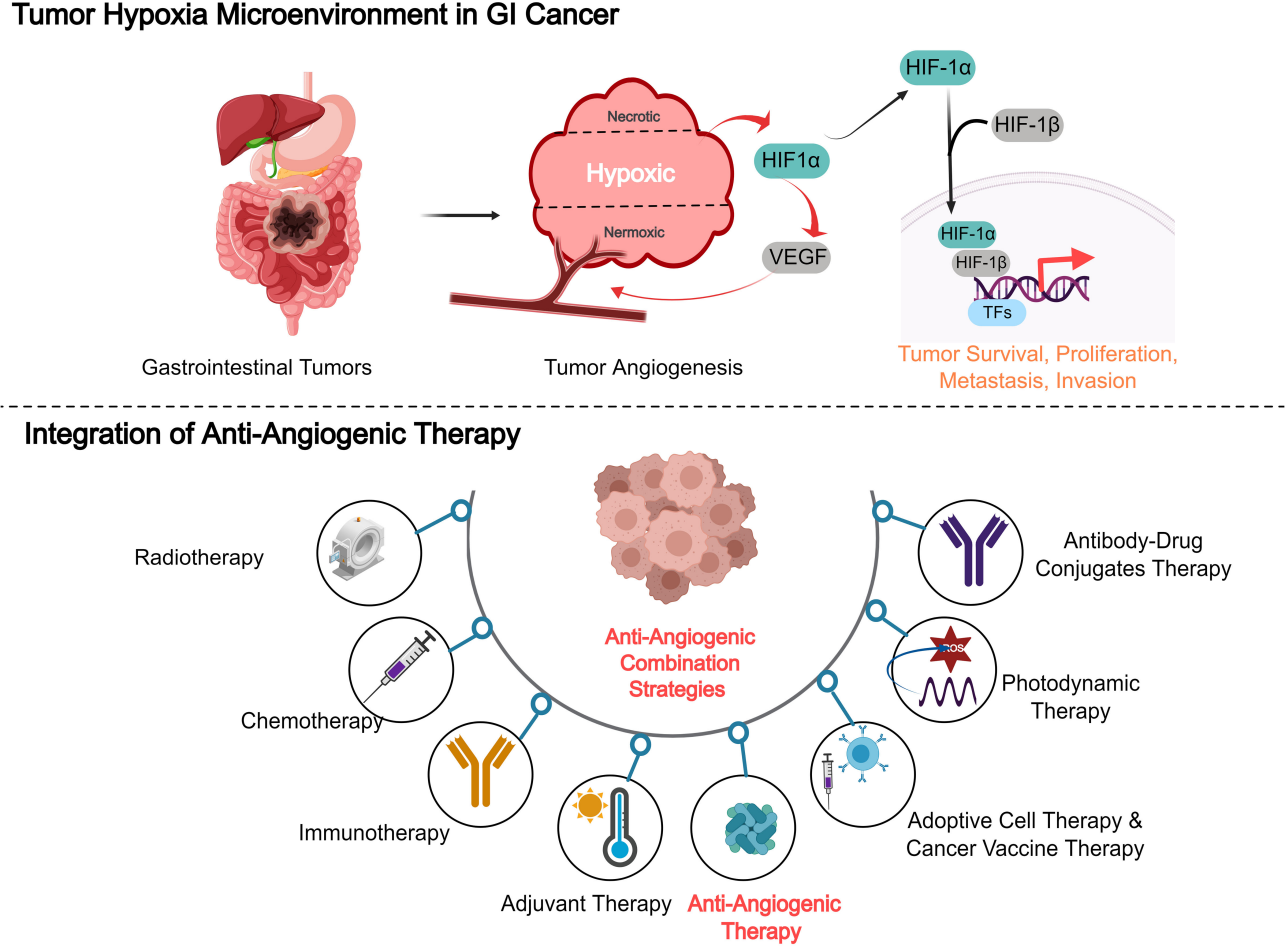
Combining anti-angiogenic therapies with other treatment modalities, such as chemotherapy, immunotherapy, and targeted therapies, in gastrointestinal cancer.

Combining anti-angiogenic therapies with other treatment modalities, such as chemotherapy, immunotherapy, and targeted therapies, has shown significant potential in enhancing therapeutic efficacy against various cancers (**Figure 3**). The rationale behind this integration lies in the complex nature of tumor biology, where single-agent therapies often fall short due to resistance mechanisms and tumor heterogeneity. For example, combining anti-angiogenic agents with immune checkpoint inhibitors has demonstrated improved anti-tumor responses by enhancing immune cell infiltration into the TME (76). Moreover, anti-angiogenesis therapies, when paired with chemotherapy, can effectively disrupt tumor adaptive responses, thereby mitigating resistance mechanisms and improving treatment efficacy. This combination has been associated with improved overall treatment outcomes, including better tumor shrinkage and a reduction in metastatic spread (79). The convergence of anti-angiogenic therapy and immunotherapy represents a paradigm shift in cancer treatment, leveraging complementary mechanisms to overcome microenvironmental resistance. Preclinical studies reveal that VEGF-mediated vascular abnormalities not only impair drug delivery but also establish an immunosuppressive niche through multiple pathways: (1) upregulation of PD-L1/CTLA-4 expression on endothelial and immune cells (80, 81), (2) recruitment of regulatory T cells and myeloid-derived suppressor cells (32), and (3) induction of hypoxia-driven adenosine accumulation (82). Anti-angiogenic agents can reverse these immunosuppressive circuits by normalizing the tumor vasculature, which improves T-cell infiltration and reprogramming myeloid cell populations (82). Clinically, anti-angiogenic agents can reverse these immunosuppressive circuits by normalizing tumor vasculature (improving T-cell infiltration) and reprogramming myeloid cell populations (83). Subsequent phase III trials in HCC (COSMIC-312) further validated this approach, showing 30-42% reductions in mortality risk compared to monotherapies (84).Furthermore, clinical trials exploring these combination strategies have demonstrated enhanced progression-free survival and reduced metastasis, particularly in aggressive cancers such as gastric and colorectal cancer (85). However, the clinical success of these combination therapies is highly contingent upon a deeper understanding of the TME and the underlying mechanisms of resistance, which can vary considerably across different cancer types. Personalized approaches that consider these factors are critical for optimizing treatment outcomes (86).

### 5.3 Clinical trial results and future prospects of combination strategies

The clinical landscape for combination strategies in anti-angiogenic therapy is rapidly evolving, with numerous trials underway to assess their efficacy and safety. Recent studies have reported promising results, indicating that combinations of anti-angiogenic agents with immunotherapy or chemotherapy can lead to enhanced therapeutic responses and improved patient outcomes. For instance, trials investigating the combination of anti-VEGF therapies with immune checkpoint inhibitors have shown increased overall survival rates in patients with advanced cancers, suggesting a synergistic effect that warrants further exploration (79). Additionally, modifiable lifestyle factors, such as diet and exercise, have been shown to influence the tumor microenvironment and may complement anti-angiogenic therapies, potentially improving patient outcomes in colorectal cancer (87). Moreover, ongoing research is focusing on identifying biomarkers that can predict responses to these combination therapies, which could facilitate personalized treatment approaches and improve clinical outcomes (76). Looking ahead, the integration of novel agents targeting specific pathways involved in angiogenesis and tumor progression, along with advancements in biomarker identification, holds great promise for enhancing the efficacy of anti-angiogenic therapies and overcoming resistance mechanisms (88). As the understanding of tumor biology deepens, the potential for innovative combination strategies to transform cancer treatment continues to expand.

## 6 Future research directions and challenges

### 6.1 Discovery and validation of novel targeted molecules

The discovery and validation of novel targeted molecules represent a significant frontier in the treatment of gastrointestinal tumors. Current therapeutic strategies often fall short due to the lack of effective biomarkers and specific therapeutic targets. Exosomes, which are nanosized vesicles that facilitate intercellular communication, have emerged as promising candidates for both biomarkers and therapeutic targets. They carry bioactive molecules, including circular RNAs (circRNAs) and long non-coding RNAs (lncRNAs), which are stably enriched and exhibit high tissue specificity. These molecules play critical roles in regulating various cellular processes such as proliferation, metastasis, and angiogenesis in gastrointestinal tumors (89). For instance, exosome circ-PVT1 has been shown to contribute to cisplatin resistance by regulating autophagy, invasion, and apoptosis via the miR-30a-5p/YAP1 axis in gastric cancer cells (90). The identification of these novel targets can enhance the precision of cancer therapies, allowing for more tailored treatment approaches that could improve patient outcomes. However, the transition from preclinical findings to clinical applications poses challenges, including the need for rigorous validation of these biomarkers in diverse patient populations and the development of effective delivery systems for therapeutic agents targeting these molecules (68).

### 6.2 Potential for personalized treatment

The potential for personalized treatment in gastrointestinal cancer is increasingly recognized, driven by advancements in genomics and molecular profiling. Personalized medicine aims to tailor treatment strategies based on individual patient characteristics, such as genetic mutations and tumor microenvironment factors. For instance, the identification of specific mutations in genes like BRAF and Rictor can guide the selection of targeted therapies, improving treatment efficacy and minimizing adverse effects (91). Furthermore, pharmacogenomic approaches can optimize drug selection and dosing, ensuring that patients receive the most effective therapies based on their unique genetic makeup (92). Despite these advancements, several challenges remain, including the need for comprehensive genomic screening in clinical settings, the integration of multi-omics data into treatment decisions, and addressing disparities in access to personalized therapies. Ongoing research is critical to refine these approaches and validate their effectiveness in improving patient outcomes.

### 6.3 Strategies to overcome drug resistance and enhance treatment efficacy

Overcoming drug resistance is a major challenge in the treatment of gastrointestinal cancers, often leading to treatment failure and poor patient prognosis. Various strategies are being explored to address this issue, including combination therapies that target multiple pathways simultaneously. Additionally, the development of nanomedicine approaches, such as liposomes and nanoparticles, offers innovative ways to deliver drugs more effectively to tumor sites while minimizing systemic toxicity (93). Furthermore, targeting the tumor microenvironment, including the stromal and immune components, is gaining attention as a strategy to mitigate resistance mechanisms. However, the complexity of tumor biology and the heterogeneity of cancer cells necessitate a multifaceted approach to develop effective strategies against resistance. Continued research into the mechanisms of resistance and the identification of novel therapeutic targets will be essential for improving treatment outcomes in gastrointestinal cancer patients.

### 6.4 Emerging technologies in angiogenesis research

Advances in single-cell RNA sequencing (scRNA-seq) and spatial transcriptomics are offering new avenues for understanding the complexities of tumor vasculature and angiogenesis (94). These cutting-edge technologies enable researchers to analyze gene expression at an unprecedented resolution, identifying novel endothelial cell subpopulations, stromal interactions, and resistance mechanisms that are crucial for angiogenesis in tumors (95). By profiling individual cells within the tumor microenvironment, scRNA-seq can uncover previously hidden cellular heterogeneity, including rare endothelial cell types that may contribute to the abnormal blood vessel formation typical of tumors (59, 96, 97). Moreover, spatial transcriptomics allows for the mapping of gene expression patterns in tissue samples while maintaining the spatial context, revealing how endothelial cells interact with surrounding stromal and immune cells (98, 99). These technologies provide valuable insights into the mechanisms of vascular normalization and resistance to anti-angiogenic therapies. For example, they can identify specific biomarkers of resistance that are associated with poor responses to current therapies, thus opening the door to the development of more targeted and effective treatments (94). As these technologies continue to evolve, they hold the potential to revolutionize the approach to anti-angiogenic therapy, enabling more precise therapeutic strategies and improving patient outcomes.

## 7 Conclusions

Targeting angiogenesis in gastrointestinal tumors represents a promising therapeutic avenue, with strategies ranging from vascular disruption to promotion. This review highlights the biological significance of angiogenesis in gastrointestinal tumor progression and the complex interplay of molecular mechanisms that regulate this process. While vascular disruption strategies such as anti-VEGF therapies have shown clinical success, challenges like resistance and adverse effects necessitate innovative approaches, including combination therapies and vascular normalization. Similarly, vascular promotion strategies, such as leveraging natural compounds and exploring the role of exosome lncRNAs and circRNAs, offer novel opportunities for enhancing tumor vascularization and drug delivery.

This article provides unique contributions by emphasizing the dual strategies of vascular disruption and normalization, a comprehensive approach that remains underexplored in the literature. The inclusion of emerging technologies, such as exosome RNAs and spatial transcriptomics, further distinguishes this review, presenting novel avenues for enhancing tumor vasculature targeting. These strategies hold great promise for not only improving the efficacy of current treatments but also overcoming key challenges such as drug resistance and the need for precise patient-specific therapies.

However, certain limitations of this review should be acknowledged. Firstly, there is a relatively limited discussion of pediatric gastrointestinal tumors, which may have distinct angiogenic characteristics compared to adult tumors and thus warrant tailored therapeutic strategies. Additionally, the cost-effectiveness of combining vascular disruption and normalization therapies remains underexplored and should be a priority in future studies to ensure that these strategies can be implemented in clinical practice without undue financial burden. Furthermore, while vascular normalization has emerged as a promising strategy, biomarkers to predict the response to normalization therapies are still not well-established (100). Thus, further research into identifying reliable biomarkers is crucial for translating vascular normalization into widespread clinical use.

Future advancements will depend on the discovery and validation of novel molecular targets, the development of personalized treatment strategies, and overcoming drug resistance through multifaceted approaches. By integrating these insights with emerging technologies and combination therapies, angiogenesis-targeted treatments hold the potential to significantly improve clinical outcomes for patients with gastrointestinal tumors. Continued research into the molecular and clinical aspects of angiogenesis will be critical for addressing the challenges of tumor heterogeneity and resistance, paving the way for more effective and precise therapeutic strategies.
